# The Effects of Alignment Quality, Distance Calculation Method, Sequence Filtering, and Region on the Analysis of 16S rRNA Gene-Based Studies

**DOI:** 10.1371/journal.pcbi.1000844

**Published:** 2010-07-08

**Authors:** Patrick D. Schloss

**Affiliations:** Department of Microbiology & Immunology, The University of Michigan, Ann Arbor, Michigan, United States of America; University of California Davis, United States of America

## Abstract

Pyrosequencing of PCR-amplified fragments that target variable regions within the 16S rRNA gene has quickly become a powerful method for analyzing the membership and structure of microbial communities. This approach has revealed and introduced questions that were not fully appreciated by those carrying out traditional Sanger sequencing-based methods. These include the effects of alignment quality, the best method of calculating pairwise genetic distances for 16S rRNA genes, whether it is appropriate to filter variable regions, and how the choice of variable region relates to the genetic diversity observed in full-length sequences. I used a diverse collection of 13,501 high-quality full-length sequences to assess each of these questions. First, alignment quality had a significant impact on distance values and downstream analyses. Specifically, the greengenes alignment, which does a poor job of aligning variable regions, predicted higher genetic diversity, richness, and phylogenetic diversity than the SILVA and RDP-based alignments. Second, the effect of different gap treatments in determining pairwise genetic distances was strongly affected by the variation in sequence length for a region; however, the effect of different calculation methods was subtle when determining the sample's richness or phylogenetic diversity for a region. Third, applying a sequence mask to remove variable positions had a profound impact on genetic distances by muting the observed richness and phylogenetic diversity. Finally, the genetic distances calculated for each of the variable regions did a poor job of correlating with the full-length gene. Thus, while it is tempting to apply traditional cutoff levels derived for full-length sequences to these shorter sequences, it is not advisable. Analysis of β-diversity metrics showed that each of these factors can have a significant impact on the comparison of community membership and structure. Taken together, these results urge caution in the design and interpretation of analyses using pyrosequencing data.

## Author Summary

Microbial communities are notoriously difficult to analyze because of their inaccessibility via culturing and high diversity. Next generation sequencing technologies have made it possible to obtain deep sampling coverage of the 16S rRNA gene; however, interpretation of the resulting data is complicated by the inability to relate sequences from variable regions within the gene to the full-length gene and ultimately, the parent genome. Here, I present a comprehensive analysis quantifying the effects of varying sequence alignment quality, pairwise distances calculation methods, sequence filtering, and regions within the 16S rRNA gene on downstream analysis using OTU- and phylogeny-based methods. This analysis indicates that each factor can have a significant effect on descriptions of α- and β-diversity. Because it is not possible to relate pyrotags to full-length 16S rRNA gene sequences directly, I encourage scientists to view pyrotags as markers within a microbiome in an analogous fashion to how geneticists view single nucleotide polymorphisms as markers within genomes.

## Introduction

The recent advent of massively-parallelized pyrosequencing platforms has enabled a renaissance in the field of microbial ecology [1], [2]. Pyrosequencing has engendered much enthusiasm since it is now possible to obtain nearly 100-times as many sequences by pyrosequencing for the same cost as using traditional Sanger sequencing technology. Although pyrosequencing is capable of generating 10^5^–10^6^ sequences per run, the sequences are between 100 and 400 bp in length. This method has become widely used among microbial ecologists to sequence PCR amplicons from variable regions within the ca. 1,500-bp 16S rRNA gene.

These massive datasets have been analyzed through the generation of phylogenetic trees [e.g. 3], assignment of sequences to operational taxonomic units (OTUs) for based on distance thresholds [e.g. 4], and classification of sequences to phylogenentic bins based on similarity to reference sequences [e.g. 5]. Each approach has received some level of evaluation using pyrotag sequencing. Liu et al. [6] asserted that phylogenies generated using pyrotags were as good as full-length sequences based on similarity of UniFrac test statistics. Several studies have evaluated various regions and methods for assigning sequences to phylotypes [7]–[9]. Finally, a recent study emphasized differences in α-diversity metrics using different regions within the 16S rRNA gene and OTU definitions [10].

Each of these studies have focused on a limited range of phylogenetic groups found in a particular environment (e.g. soil, mouse cecum, human feces) and have glossed over more fundamental questions related to how alignment quality, methods of calculating pairwise genetic distances, sequence filtering, and region affects downstream analysis and their relationship to full-length sequences. Alignment quality is expected to significantly affect pairwise distances. Investigators have either used reference alignments to align sequences that implicitly incorporate the secondary structure of the 16S rRNA molecule [11]–[14] or they have used methods that do not consider the secondary structure [15], [16]. Previous results have shown that the manually-curated SILVA reference alignment provides superior complementary base-pairing within the secondary structure compared to the greengenes alignment, which appears haphazard; the RDP alignment does not align the variable regions [11]. Considering the focus of these studies is on the variable region, there is the added complication that these areas are difficult to align accurately. To overcome limitations in alignment of variable regions, many studies have employed the use of masks to filter the troublesome regions [e.g. 3]. Yet, these filters remove a considerable amount of information from already information-sparse data (Table 1). The actual method of calculating distances is also typically taken for granted. Practically every 16S rRNA survey has made use of substitution models that assume that an alignment gap represents missing data instead of a mutation [e.g. 17]. The decision to use such a model seems motivated more by a sense of phylogenetic guilt than by biology. It is also unknown how distances calculated between partial sequences predict distances between full-length sequences. To make data analysis more tractable, some have employed heuristics based on correlations between kmer- and sequence-based pairwise distances to select which pairs of sequences to align and group within OTUs [16]. It is unclear how these correlations vary across regions within the 16S rRNA gene or what the level of risk is for falsely ignoring pairs of similar sequences. Finally, most studies make the implicit assumption that distances between partial sequences are not significantly different from those of full-length sequences; however, this is a questionable assumption as it is well-established that the 16S rRNA gene does not evolve uniformly along its length. This is apparent in the choice a 3% distance cutoff, which is used as a proxy species definition for full-length sequences, to define species using sequences from variable regions [e.g. 2], [4]. Each of these factors is expected to have a significant effect on the analysis, interpretation, and generalizability of 16S rRNA gene surveys.

**Table 1:** Summary of the 13 regions within the 16S rRNA gene that were used in this study.

Region	*E. coli* numbering	Platform	Example Ref.	Masking	Average number of bases^a^
**V19**	2–1491	Sanger^b^	[32]	None	1454 (1399–1490)
				Lane	1255 (1244–1256)
**V12**	28–337	Titanium^c^		None	306 (276–332)
				Lane	239 (238–239)
**V13**	28–514	Titanium	HMP^f^	None	480 (428–508)
				Lane	386 (384–386)
**V14**	28–784	Sanger	[17]	None	750 (698–779)
				Lane	656 (653–656)
**V2**	100–337	FLX^d^	[3]	None	240 (223–257)
				Lane	198 (197–198)
**V23**	100–514	Titanium		None	415 (378–437)
				Lane	345 (343–345)
**V3**	357–514	FLX/Illumina^e^	[33]	None	158 (135–161)
				Lane	128 (127–128)
**V35**	357–906	Titanium	HMP	None	546 (523–552)
				Lane	507 (504–507)
**V4**	578–784	FLX	[8]	None	207 (206–208)
				Lane	207 (206–207)
**V6**	986–1045	FLX/Illumina	[5]	None	60 (57–66)
				Lane	27 (27–27)
**V69**	986–1491	Titanium	HMP	None	507 (489–516)
				Lane	411 (407–412)
**V89**	1100–1491	Titanium		None	392 (373–403)
				Lane	330 (326–331)
**V9**	1300–1491	FLX	[4]	None	192 (182–197)
				Lane	170 (146–147)

Each sub-region was generated from the sequences in the V19 database.

a The numbers in the parentheses represent the 95% confidence interval.

b Sanger reads are estimated to be up to 800 bp and can be used to sequence the same molecule multiple times.

c GS FLX Titanium reads average ∼400 bp (amplicon kit released 11/2009).

d GS FLX reads average ∼240 bp.

e Illumina reads average ∼200 bp.

f The NIH Human Microbiome Project is considering these regions for their cross-sectional studies.

Here, I used a collection of full-length 16S rRNA gene sequences representing 43 bacterial phyla to quantify how alignment quality, distance calculation methods, masking, and region within the 16S rRNA gene affect out ability to assess α- and β-diversity. The results of these analyses urge greater caution in how surveys are designed and interpreted.

## Results

### The effect of alignment on genetic distances

For each of the 13 regions I used various alignment methods to calculate 91,131,750 pairwise distances assuming that a series of consecutive gaps represented one insertion or deletion. The SILVA, greengenes, and RDP alignments represent a gradation in the level of attention given to aligning the variable regions and are each guided by the secondary structure of the 16S rRNA gene. In contrast, the MUSCLE and pairwise alignments are attempts to optimize the alignment between sequences based on a limited number of parameters that are set *a priori*. To compare the pairwise distances calculated for the same pairs of sequences across alignments, I calculated the regression coefficients describing the relationship between the distances for the greengenes, RDP, MUSCLE, and pairwise alignments and the SILVA alignment for each region (Table 2). Distance calculations for this analysis assumed that consecutive gap positions were the product of a single insertion or deletion mutation (i.e. one gap). With the exception of the V3 and V4 regions, the RDP alignment for each of the regions predicted greater genetic diversity than that of the SILVA alignment. Interestingly, the greengenes alignment, which does a poor job of aligning the variable regions, predicted between 9 and 33% more genetic diversity for each region than the RDP alignment, which does not attempt to align the variable regions. Visual inspection of the greengenes alignment suggests that in many instances the variable region alignments are somewhat random [11]. I observed that the MUSCLE-generated alignments described considerably greater genetic diversity than any of the other methods for the V3, V6, and V9 regions (Table 2); however, the use of pairwise alignments yielded smaller distances than those calculated with the other alignment methods because pairwise alignment methods optimize the alignment without the constraint of preserving positional homology across multiple sequences (Table 2). Perhaps most worrisome is the observation that with the exception of the distances calculated from pairwise alignments, regressions of the other alignment methods to the SILVA-based alignment typically did a poor job of accounting for the variation in the distances (Table 2). These data make it clear that variation in alignment quality can have a significant impact on the genetic diversity that is calculated between the same pairs of sequences.

**Table 2:** Slope coefficients and R^2^ values for the comparison of one gap distances calculated for SINA-aligned sequences extracted from different regions within the 16S rRNA gene sequence to one gap distances calculated using sequences aligned by different methods.

Region	Statistic	RDP	greengenes	MUSCLE	Needleman
**V19**	Slope	1.06	1.17	1.04	0.93
	R^2^	0.97	0.77	0.98	0.99
**V12**	Slope	1.13	1.25	1.11	0.93
	R^2^	0.80	0.52	0.77	0.91
**V13**	Slope	1.08	1.20	1.07	0.93
	R^2^	0.88	0.62	0.92	0.93
**V14**	Slope	1.06	1.16	1.05	0.94
	R^2^	0.94	0.74	0.96	0.97
**V2**	Slope	1.04	1.21	1.16	0.97
	R^2^	0.94	0.67	0.64	0.95
**V23**	Slope	1.04	1.18	1.09	0.95
	R^2^	0.96	0.74	0.92	0.96
**V3**	Slope	1.00	1.11	2.07	1.02
	R^2^	0.91	0.67	0.14	0.96
**V35**	Slope	1.01	1.12	1.04	0.95
	R^2^	0.98	0.83	0.97	0.98
**V4**	Slope	1.00	1.09	1.08	0.98
	R^2^	0.99	0.77	0.87	0.98
**V6**	Slope	1.09	1.42	3.04	0.98
	R^2^	0.66	0.14	0.30	0.97
**V69**	Slope	1.07	1.21	1.10	0.94
	R^2^	0.92	0.70	0.91	0.97
**V89**	Slope	1.05	1.18	1.12	0.96
	R^2^	0.94	0.78	0.86	0.98
**V9**	Slope	1.08	1.19	1.58	0.96
	R^2^	0.80	0.67	0.36	0.96

### Effect of alignment on interpretation of α-diversity

**Figure 1:**
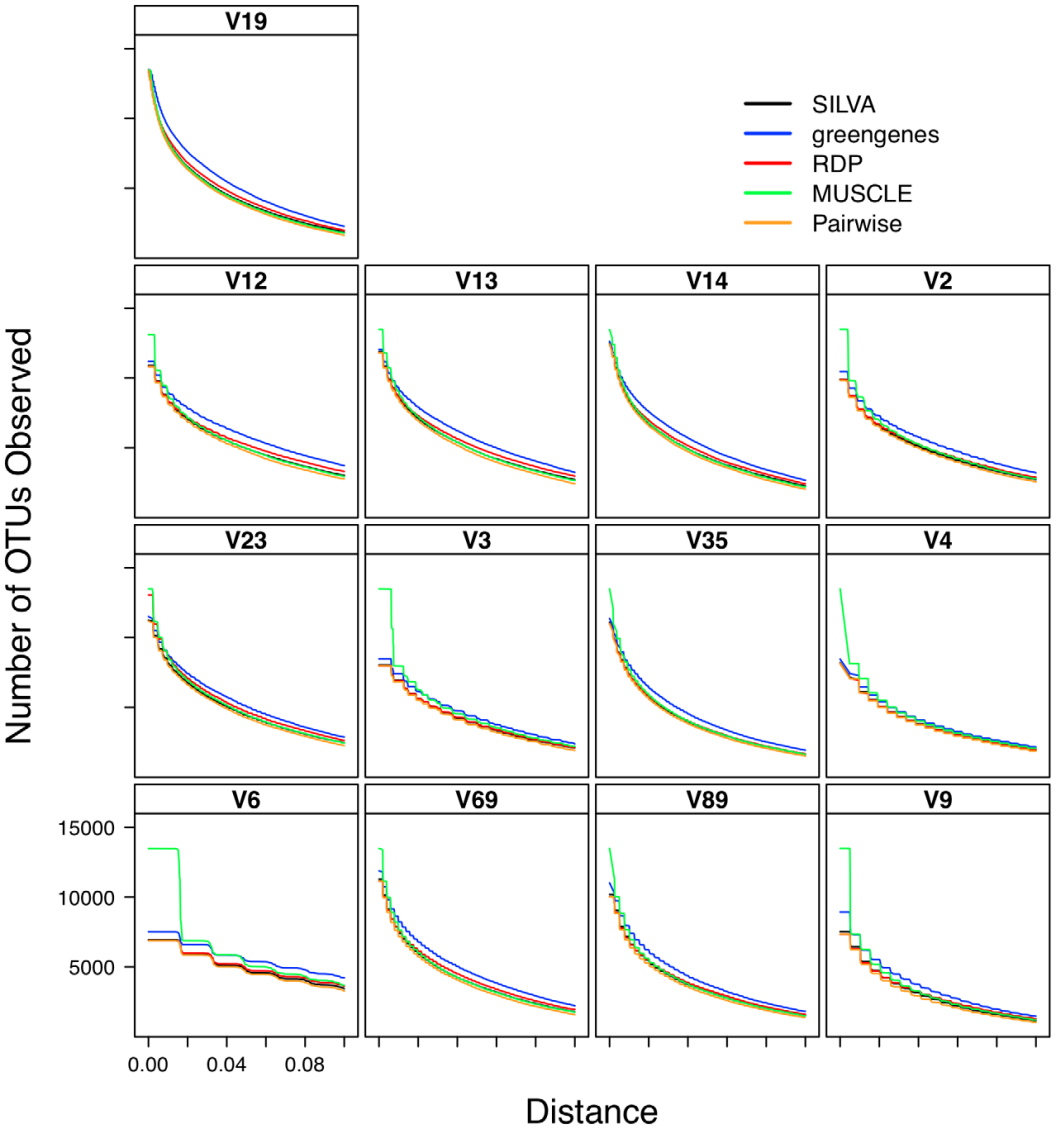
The number of OTUs observed as a function of genetic distance for various regions within the 16S rRNA gene when using different sequence alignments.

**Figure 2:**
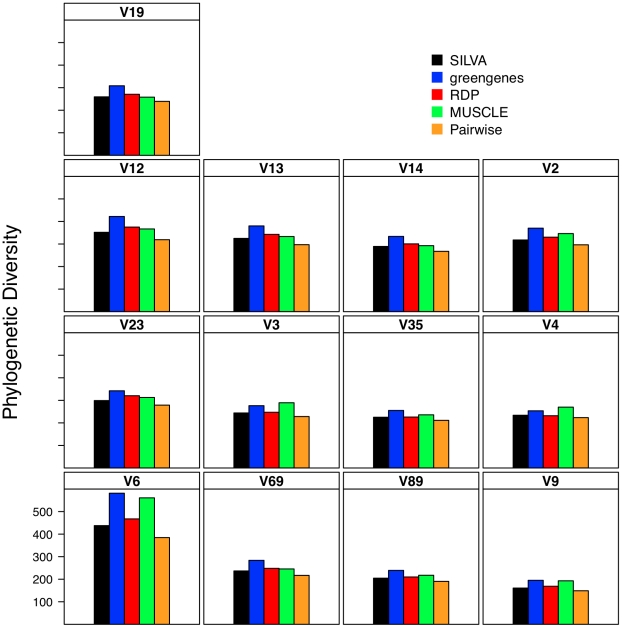
The phylogenetic diversity observed for different regions within the 16S rRNA gene when using different alignments. Phylogenetic diversity was measured by calculating the total branch length for a phylogenetic tree.

Considering the poor correlation between the distances generated from the five alignment methods, it was necessary to determine the effect of this variation on the ability to accurately describe and compare communities. As expected based on the genetic distance analysis, the number of OTUs observed using the greengenes alignment was routinely higher than that observed using the other alignment methods and the number of OTUs observed using the pairwise alignment method was routinely the lowest (Fig. 1). Inspection of these lineage through time plots identified a stair-like appearance for many of the regions. This was due to the loss of information as sequence length decreased. The most extreme example of this phenomenon was for the V6 region that had an average sequence length of 60 bp. Each difference between a pair of V6 sequences changed the distance by approximately 0.0167 units, which is the step-length observed for the V6 data in Fig. 1. When the phylogenetic diversity of the datasets was calculated, the greengenes aligned sequences had the highest phylogenetic diversity and the pairwise aligned sequences had the lowest (Fig. 2). One limitation of the phylogenetic diversity metric is that it is difficult to interpret the statistic and so it is unclear how biologically meaningful the level of variation observed is in Fig. 2.

### Effect of alignment on interpretation of β-diversity

**Figure 3:**
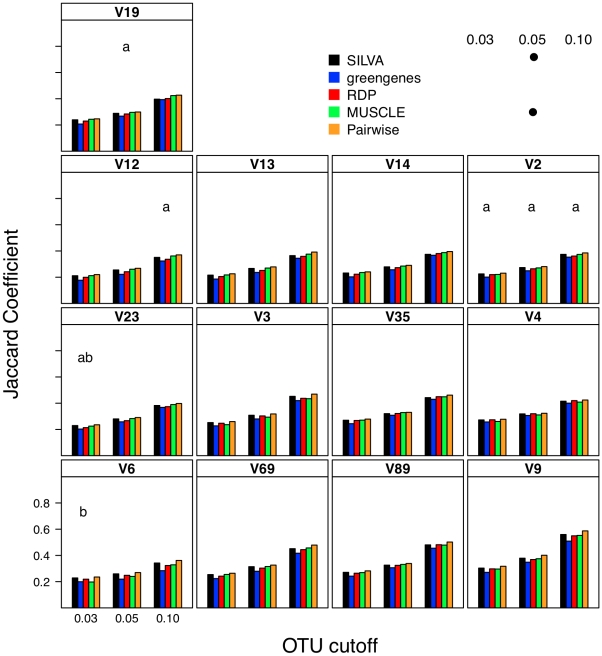
The Jaccard coefficient calculated between two mock communities (described in Materials & Methods) for different OTU definitions and alignments. Each bar represents the average coefficient value for 100 randomized partitionings of the data. Within the same OTU cutoff, alignment strategies with the same symbol and regions with the same letter were not significantly different from each other.

**Figure 4:**
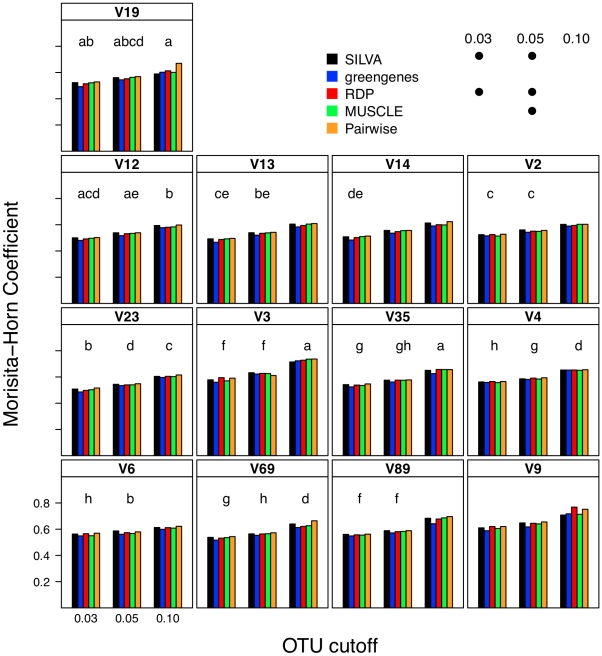
The Morisita-Horn coefficient calculated between two mock communities (described in Materials & Methods) using different OTU definitions and alignments. Each bar represents the average coefficient value for 100 randomized partitionings of the data. Within the same OTU cutoff, alignment strategies with the same symbol and regions with the same letter were not significantly different from each other.

**Figure 5:**
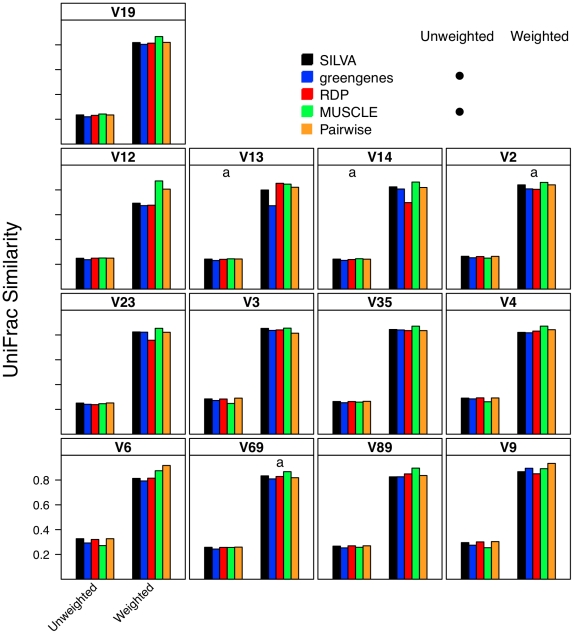
Unweighted and weighted UniFrac similarity values calculated between two mock communities (described in Materials & Methods) using different alignments. Each bar represents the average coefficient value for 100 randomized partitionings of the data. Within the same UniFrac approach, alignment strategies with the same symbol and regions with the same letter were not significantly different from each other.

To describe β-diversity, I used two OTU-based metrics (Figs. 3 and 4) and two phylogenetic-based metrics (Fig. 5) to measure the sensitivity of the metrics to alignment quality. Sequences were partitioned so that they would represent two samplings of communities whose Jaccard similarity index was 0.80, but whose Morisita-Horn similarity index was 0.60 with a cutoff of 0.05 when defining OTUs with full-length sequences. Because the sampling of the two simulated communities was limited (ca. 6,750 sequences per community), the Jaccard and unweighted UniFrac statistics did not equal the expected values. Within this simulation framework, the effect of alignment was generally highly statistically significant across metrics of β-diversity (p≪0.001); however it is unclear how biologically meaningful the observed differences were.

### The effect of distance calculation method on genetic distances

Using the same SILVA-aligned sequences that I analyzed above, I investigated the effect of different distance calculation methods on downstream analyses. Specifically, I considered the one gap calculator (i.e. a gap of any length between two sequences represents a single mutation) and each gap (i.e. gaps length *n*, represent *n* mutations) and ignore gap calculators (i.e. gapped characters are not considered in calculating a distance; Table 3). The slope of lines forced through the origin indicated that the each gap calculator calculated between 0 (V4) and 9% (V3) more genetic diversity than the one gap calculator. With the exception of the V3 region (69%), the regression between the each gap and one gap calculators accounted for more than 87% of the variation in the distances. The differences in the explanatory power of the regression were a function of frequency of gaps longer than 1 nucleotide. The ignore gap calculator calculated between 2 (V4) and 7% (V9) less genetic diversity than the one gap calculator. The regression between the ignore gap and one gap calculators accounted for more than 94% of the variation in the data. Until there is a more well-developed theoretical basis for selecting a method for treating gaps in sequence alignments, these results suggest that treating gaps of any length as a single mutation is a middle ground between ignoring them and treating each of them as a separate evolutionary event.

**Table 3:** Slope and R^2^ values for the regression of one gap pairwise distances onto each gap, ignore gap, Lane mask-filtered one gap, and kmer distances using SINA-aligned sequences over the same region.

Region	Statistic	Each gap	Ignore gaps	Filtered	kmer
**V19**	Slope	1.02	0.94	0.66	3.73
	R^2^	0.99	0.99	0.89	0.97
**V12**	Slope	1.02	0.94	0.56	3.91
	R^2^	0.87	0.97	0.47	0.89
**V13**	Slope	1.02	0.94	0.55	3.80
	R^2^	0.92	0.97	0.56	0.91
**V14**	Slope	1.02	0.95	0.67	3.87
	R^2^	0.96	0.98	0.75	0.95
**V2**	Slope	1.01	0.97	0.71	4.31
	R^2^	0.90	0.99	0.58	0.92
**V23**	Slope	1.01	0.96	0.64	3.99
	R^2^	0.92	0.98	0.66	0.93
**V3**	Slope	1.09	0.95	0.77	4.69
	R^2^	0.69	0.94	0.52	0.83
**V35**	Slope	1.01	0.97	0.79	4.24
	R^2^	0.99	0.98	0.84	0.94
**V4**	Slope	1.00	0.98	0.99	4.70
	R^2^	1.00	0.98	0.99	0.93
**V6**	Slope	1.00	0.96	1.17	4.82
	R^2^	1.00	0.95	0.38	0.86
**V69**	Slope	1.02	0.94	0.73	3.94
	R^2^	0.98	0.98	0.79	0.94
**V89**	Slope	1.03	0.93	0.85	4.35
	R^2^	0.94	0.97	0.86	0.95
**V9**	Slope	1.01	0.93	0.76	4.60
	R^2^	0.95	0.94	0.69	0.91

Pairwise kmer distances were much larger than the alignment-based calculators and their regression onto the one gap calculated distances accounted for between 83 and 97% of the variation observed between the distances. In order to have no risk of falsely ignoring true one gap pairwise distances smaller than 0.10, it was necessary to keep kmer distances smaller than 0.45 (V19) to 0.73 (V6). This would result in needing to calculate between 3.3- and 9.1-fold more distances than would be needed by alignment-based methods.

### Effect of distance calculation method on interpretation of α-diversity

**Figure 6:**
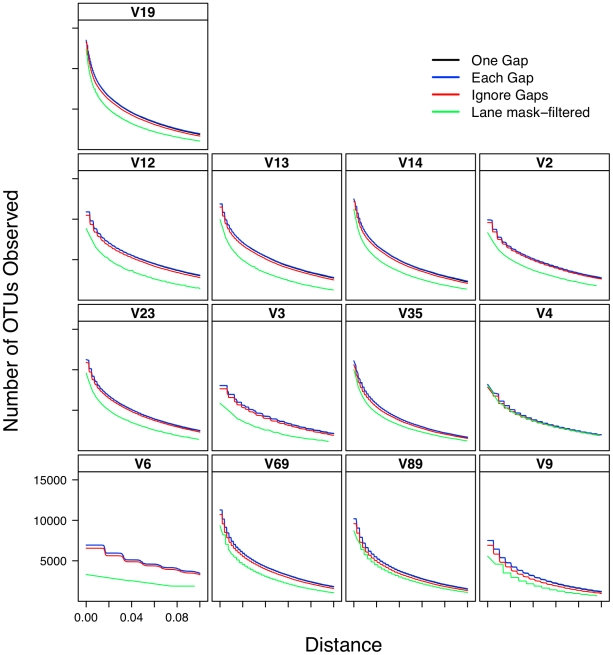
The number of OTUs observed as a function of genetic distance for various regions within the 16S rRNA gene when using different methods of calculating distances and masking sequences.

**Figure 7:**
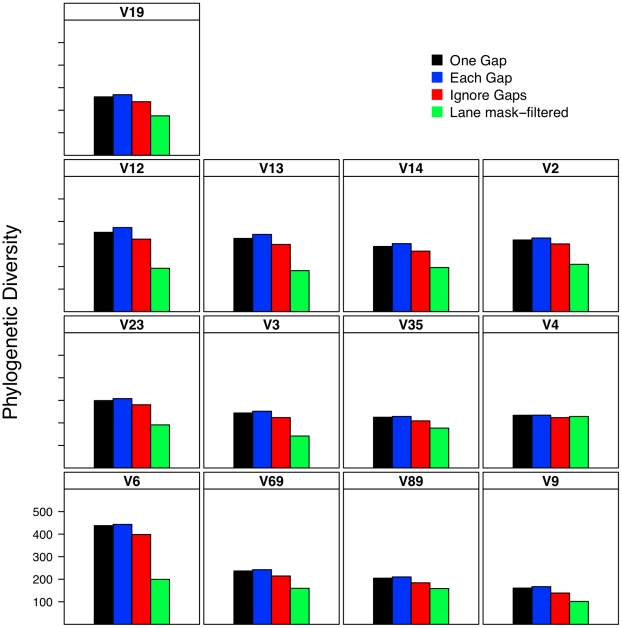
The phylogenetic diversity observed for different regions within the 16S rRNA gene when using different methods of calculating distances and masking sequences. Phylogenetic diversity was measured by calculating the total branch length for a phylogenetic tree.

Lacking a theoretical basis for treating gaps as a single evolutionary event, I was curious how much measures of α- and β-diversity are affected by the choice of a distance calculator. I used an OTU-based approach to determine the effect of distance calculation methods on the richness of OTUs within the dataset (Fig. 6) and a phylogeny-based approach using total branch length to measure phylogenetic diversity (Fig. 7). As would be predicted, the number of observed OTUs at any genetic distance was greatest with the each gap and least with the ignore gap calculators; the one gap and each gap calculators generated comparable numbers of OTUs. When I analyzed the effect of region and distance calculation method on the phylogenetic diversity of the datasets, there were qualitative trends between methods and regions that could have been predicted from the regression analysis in Table 2 (Fig. 7). These analyses suggest that the difference observed in α-diversity when using either the one gap or each gap calculator is unlikely to be biologically meaningful.

### Effect of distance calculation method on interpretation of β-diversity

**Figure 8:**
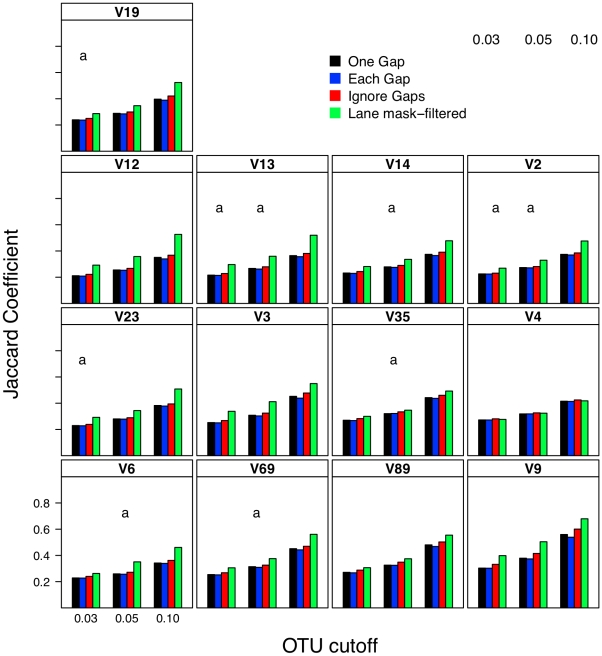
Jaccard similarity values calculated between two mock communities (described in Materials & Methods) for different OTU definitions, methods of calculating distances, and masking sequences. Each bar represents the average coefficient value for 100 randomized partitionings of the data. Within the same OTU cutoff, regions with the same letter were not significantly different from each other; for each OTU cutoff all distance calculation methods were significantly different from each other.

**Figure 9 pcbi-1000844-g009:**
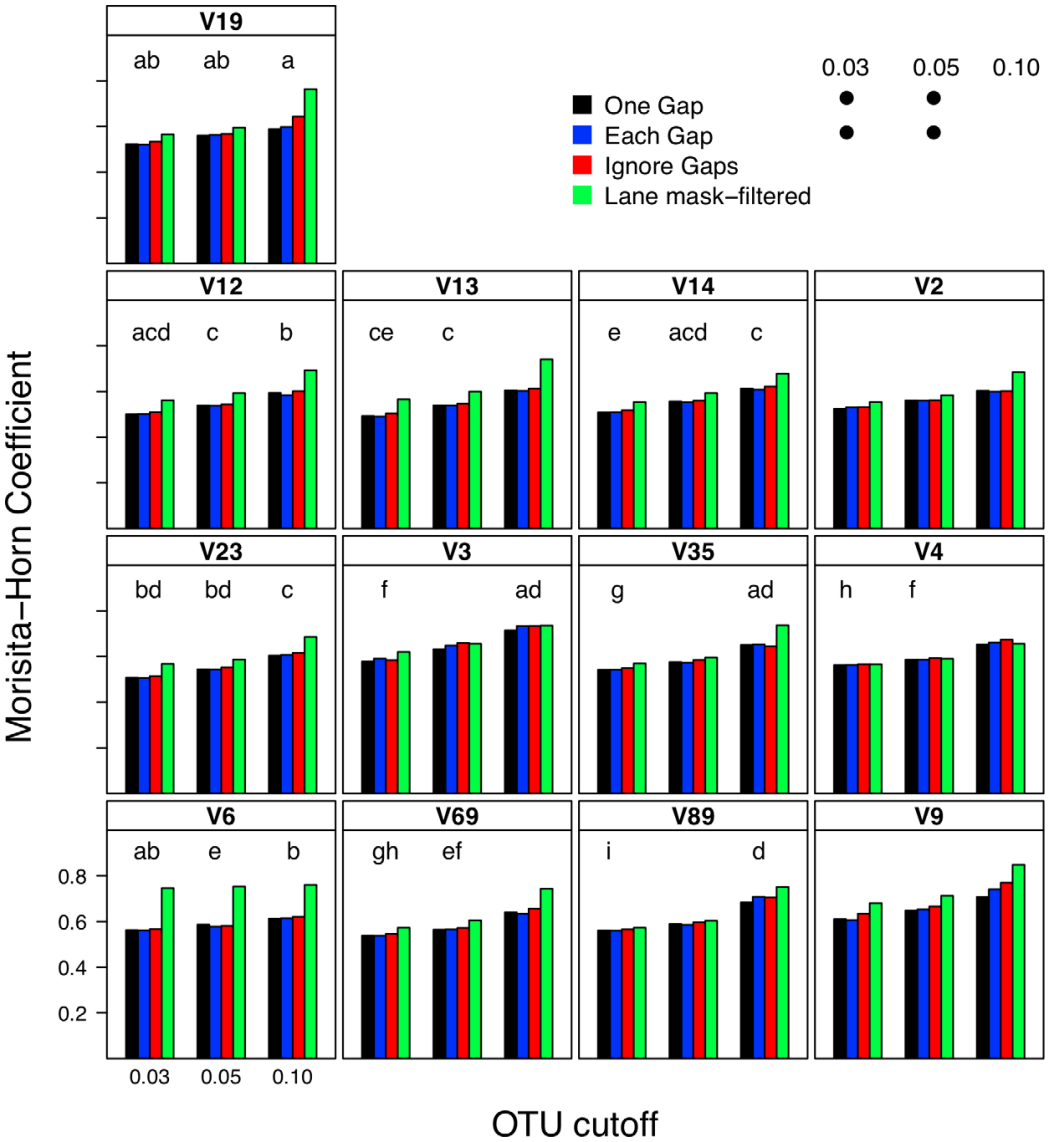
The Morisita-Horn coefficient calculated between two mock communities (described in Materials & Methods) using different OTU definitions, methods of calculating distances, and masking sequences. Each bar represents the average coefficient value for 100 randomized partitionings of the data. Within the same OTU cutoff, distance calculation methods with the same symbol and regions with the same letter were not significantly different from each other.

**Figure 10:**
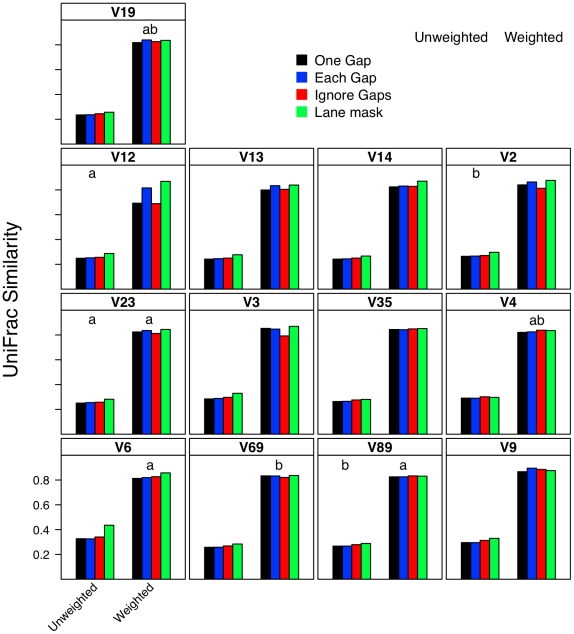
Unweighted and weighted UniFrac similarity values calculated between two mock communities (described in Materials & Methods) using different methods of calculating distances and masking sequences. Each bar represents the average coefficient value for 100 randomized partitionings of the data. Within the same UniFrac method, regions with the same letter were not significantly different from each other; for both UniFrac methods the distance calculation methods were all significantly different from each other.

I next investigated what effect each calculator method had on two OTU-based (Figs. 8 and 9) and two phylogeny-based β-diversity measures (Fig. 10). For the OTU-based metrics, ignoring gaps resulted in an over-estimate of the similarity between the two communities and counting each gap resulted in an under-estimate. Increasing and decreasing the cutoff used to define the OTUs had a parallel effect on the Jaccard and Morisita-Horn indices (Figs. 8 and 9). These results occurred because ignoring gaps and increasing the threshold each dampen the differences between sequences and pull more sequences into an OTU so that more OTUs are likely to be shared; the same phenomenon was observed when sequences were filtered using the Lane mask (see below). Penalizing each gap or making the OTU definition more stringent had the opposite effect. The calculated Jaccard coefficients were not significantly different between the one gap and each gap distance calculation methods when using the 0.03 and 0.05 OTU cutoffs (Fig. 8); all four distance calculation methods yielded statistically significant differences in Morisita-Horn coefficients, regardless of the OTU cutoff. For the phylogeny-based methods, the observed differences between each of the distance calculation methods were statistically significant (Fig 10). Although the differences between distance calculation methods were highly statistically significant (p≪0.001), it is unclear how biologically meaningful the differences were.

### Effects of filtering sequences using the Lane mask on analysis

To circumvent alignment quality problems, the Lane mask has been used to filter variable regions from 16S rRNA genes. Results of analyses using filtered sequences aligned by any method or when distances were calculated by any method did not vary to a meaningful degree. Comparison of distances calculated using filtered sequences to those calculated using unfiltered sequences showed that filtering significantly reduced the genetic diversity observed between sequences (Table 3). With the exception of the V4 and V6 regions, masking removed between 15 and 45% of the genetic diversity. The V4 region is largely unaffected by the Lane mask and the average length of V6 sequences following the Lane mask treatment was only 27 bp, which made the resulting pairwise distances of dubious value (Table 1). As would be expected, the number of OTUs and phylogenetic diversity observed using Lane mask-filtered sequences was significantly lower than those calculated with the unfiltered sequences. For the four β-diversity measures, when the Lane mask-filtered sequences were analyzed, the communities appeared more similar than for non-filtered SILVA-aligned sequences (Figs. 8–10). One explanation for this observation is that because filtering makes sequences more similar to each other, it also makes communities appear more similar to each other. Although useful for broad-scale phylogenetic analysis at the level of a kingdom or phylum, filters remove the sequence information necessary to differentiate populations within a community. Ultimately, application of such filters is troublesome because it mutes the signals that differentiate communities.

### Relationship between the genetic diversity calculated between full-length and regional sequences

I compared the one gap distances calculated for each of the 12 regions from each alignment to the one gap distances calculated from the full-length SILVA alignments (Table 4). The regression of pairwise distances calculated from a sub-region onto distances calculated from full-length sequences was rarely near 1.00. The most extreme case was the V6 region for which distances were nearly 3-fold higher than distances calculated using full-length sequences. Conversely, sequences from the V9 region were 33% less diverse than their full-length counterparts. In general, genetic diversity decreased along the length of the 16S rRNA gene. Although one could use these regression coefficients to relate data collected from one region to that from full-length sequences, the ability of the regression to explain the variation observed between sub-region and full-length sequences was quite poor. As expected, longer regions did the best job of relating the variation between sub-regions and full-length sequences. For example, when using the SILVA alignments, the regression of the V14, V35, and V69 distances onto the full-length distances accounted for 87, 77, and 77% of the variation in distances. Shorter regions such as the V3, V6, and V9 accounted for 26, 36, and 46% of the variation (Table 4). This analysis revealed that all sub-regions are limited in their capacity to serve as surrogates for full-length 16S rRNA gene sequences.

**Table 4:** Regression coefficients and R^2^ values for the comparison of one gap distances calculated for different regions within the 16S rRNA gene sequence and aligned by different methods to the one gap distances calculated using SINA aligned full-length sequences.

Region	Statistic	SILVA	greengenes	RDP	MUSCLE	Needleman
**V19**	Slope	NA	1.17	1.06	1.04	0.93
	R^2^	NA	0.77	0.97	0.98	0.99
**V12**	Slope	1.50	1.79	1.65	1.74	1.36
	R^2^	0.70	0.55	0.64	0.57	0.70
**V13**	Slope	1.31	1.52	1.40	1.40	1.19
	R^2^	0.73	0.60	0.66	0.74	0.72
**V14**	Slope	1.13	1.29	1.19	1.18	1.05
	R^2^	0.87	0.72	0.83	0.88	0.87
**V2**	Slope	1.39	1.61	1.43	1.57	1.31
	R^2^	0.70	0.59	0.69	0.62	0.70
**V23**	Slope	1.21	1.38	1.26	1.29	1.13
	R^2^	0.73	0.64	0.71	0.72	0.74
**V3**	Slope	1.05	1.15	1.06	1.63	0.97
	R^2^	0.26	0.26	0.24	0.23	0.27
**V35**	Slope	0.90	0.99	0.90	0.93	0.85
	R^2^	0.77	0.68	0.76	0.75	0.76
**V4**	Slope	0.97	1.05	0.97	1.05	0.94
	R^2^	0.57	0.50	0.56	0.55	0.57
**V6**	Slope	2.98	3.52	3.30	4.99	2.62
	R^2^	0.36	0.31	0.34	0.24	0.37
**V69**	Slope	0.98	1.16	1.05	1.04	0.90
	R^2^	0.77	0.62	0.72	0.77	0.78
**V89**	Slope	0.79	0.93	0.84	0.86	0.75
	R^2^	0.70	0.62	0.67	0.68	0.70
**V9**	Slope	0.67	0.83	0.76	0.85	0.63
	R^2^	0.46	0.43	0.42	0.39	0.46

### Relationship between sub-region differences and differences in α-diversity

The distance-based analysis clearly showed significant differences between distances calculated from sub-regions and full-length sequences. The OTU-based analysis in Fig. 1 demonstrates that there was a clear difference in the number of OTUs observed across regions for a given genetic distance as well as the level of curvature observe observed in the lineage-through-time plots (Figs. 1 and 6). In the phylogenetic-based analysis those regions that described more genetic diversity than the full-length sequences had greater phylogenetic diversity than the phylogenetic diversity calculated for the full-length sequences whereas the regions that described less genetic diversity yielded greater phylogenetic diversity (Figs. 2 and 7).

### Relationship between sub-region differences and differences in β-diversity

Using pyrotag data introduces several complexities to β-diversity analyses. Moving across regions, but using the same OTU definition could lead one to overestimate community similarity. For example, the average Morisita-Horn similarity for full-length SILVA-aligned sequences with one gap distances was 0.56. Using similarly treated sequences from the V12, V13, V14, and V23 regions I calculated Morisita-Horn values between 0.57 and 0.60; however those from the other 8 regions yielded values between 0.64 (V2) and 0.79 (V9). For a single region, changing the OTU cutoff also had a significant effect on the Morisita-Horn index. For instance, full-length SILVA-aligned sequences yielded 0.52, 0.56, and 0.86 for cutoffs of 0.03, 0.05, and 0.10. This spread in Morisita-Horn values between the 0.03 and 0.10 OTU cutoffs (0.34) was the largest of any region. The narrowest spread was observed for the V6 region (0.06). In contrast to the Morisita-Horn values, there was little variation in the unweighted or weighted UniFrac statistic when comparing sequences analyzed by the same alignment and distance calculation method. With the exception of the V6 region (0.33), the average unweighted UniFrac values varied between 0.24 (V13, V14, V19) and 0.30 (V9) and with the exception of the V12 region (0.69), the average weighted UniFrac values varied between 0.80 (V13) and 0.87 (V9); the value for the full-length sequence was 0.82. Similar to the α-diversity measure of phylogenetic diversity, an added complication of phylogeny-based methods is the complexity of interpreting the proportion of branch length that is shared between or unique to two communities and how such proportions relate to classical β-diversity measures. Thus, it is difficult to interpret the biological significance of such variation. Regardless, the results of the OTU- and phylogeny-based analyses demonstrate that caution must be taken in extrapolating results from one region to another.

## Discussion

The ability to define OTUs and reconstruct phylogenies allows an investigator to approach their problem using the data as they present themselves without being confined to an *a priori* taxonomy. Regardless, the analysis I have presented indicates that comparing results obtained by sequencing one region of the 16S rRNA gene can not be easily compared to those obtained using full-length sequences. Ultimately, the fact that the 16S rRNA gene does not evolve uniformly across its length complicates its analysis. Technical limitations require investigators to select a region based on the availability of conserved PCR primers, fragment length, and the ability to generate high quality sequence. Analytical limitations require investigators to select a region based on the availability of database sequences for that region, the ability to accurately classify sequences, and the level of genetic diversity found in the region. Until there is a standardized approach, individual investigators will continue to select different regions for their analysis. Studies such as this are necessary to inform investigators about the strengths and weaknesses of the various regions within the 16S rRNA gene. Based on this analysis, it is clear that regardless of the region, longer reads will improve one's ability to relate their analysis to full-length sequences. As sequence lengths increase to the point that pyrosequencing full-length 16S rRNA genes is possible, this discussion will be unnecessary. Ultimately, all pyrotag regions represent a marker of a marker of genomic diversity. Even if full-length 16S rRNA gene sequencing is possible, it is still just a marker of genomic diversity. Correlations between the complete genome sequence and full-length 16S rRNA gene sequences are probably just as poor as correlations between full-length 16S rRNA gene sequences and their sub-regions [e.g. 18]. Although one may endeavor to characterize and compare the composition of multiple communities, any cutoffs that are employed are at best empirical and hopefully have some biological meaning.

I have shown that alignment quality has a significant impact on downstream data analysis. Because the 16S rRNA gene sequence follows a well-determined secondary structure, it is possible to objectively state that one alignment is better than another. Furthermore, pairwise and multiple sequence alignments that ignore the secondary structure are unadvisable on theoretical grounds. Such methods are also unadvisable on technical grounds as the time and memory required to complete them typically scales in excess of the number of sequences squared; the time required to perform a profile-based alignment scales linearly with the number of sequences.

A significant factor in the analysis of DNA sequences is the calculation of pairwise distances. The rich literature developed for protein-coding sequences has generated the Jukes-Cantor, Kimura, Hasegawa-Kishino-Yano and other substitution models [reviewed in 19]. Yet these models ignore gapped positions, which I have shown to have a significant impact on downstream analyses. Substitution models for structural RNA molecules such as the 16S rRNA gene are not well developed or widely used [20], [21]. It is underappreciated that use of short sequence or filtering methods such as the Lane mask reduces the precision and information represented by a distance. For instance, if there are fewer than 200 bases being considered, then it is difficult to place much confidence in an OTU threshold of 0.03 (i.e. 6 differences) when one considers the potential impact of PCR, sequencing, and alignment artifacts. Furthermore, reducing the information content of a 1,500 bp molecule to a 200-bp sequence read will affect the confidence placed in the generation of phylogenetic trees and OTU assignments. Althoguh removing non-informative positions can be helpful for reconstructing broad phylogenies, the α- and β-diversity analyses described here are adversely affected by removing this fine level sequence diversity. These are clearly issues that warrant further attention.

This study has ramifications on how analyses are performed. Since it is clear that the 16S rRNA gene does not evolve uniformly across its length, it is critical that sequences fully overlap before they are compared. For example, consider an analysis that includes sequences from the V2 region and those from the V12 region. The V12 sequences will have higher pairwise distances amongst each other than compared to the V2 region because the V1 region is evolving at a faster rate. Thus, the comparison of short and long sequence reads will add artifacts into the analysis, which will overstate the richness within the community. Although not explored here, it is likely that similar problems will be encountered in analyses where a taxonomy hierarchy is used to assign sequences to bins. Thus it is critical that sequences are trimmed to start and end at the same sequence-based landmarks. Because pyrosequencing does not yield a uniform length sequence read, this introduces a conundrum of whether to favor fewer long reads or many short reads. Because it is impossible to compare pyrotags to the full-length sequences accurately, it seems appropriate to increase the power of other statistical analyses by sacrificing sequence length in favor of having more sequence reads.

Next generation sequence analysis of 16S rRNA genes offers the first opportunity to replicate analyses, develop more complex experimental designs, and to increase sampling depth and breadth. The results of this study encourage one to see pyrotags as markers within a metagenome and suggest a different way of considering microbial community analysis. Just as single nucleotide polymorphisms (SNPs) have been used as markers of disease in genome-wide association studies (GWAS), which may have no direct effect on a genes phenotype, pyrotags no doubt will serve as a useful analog to SNPs for the nascent field of metagenome-wide association studies (MWAS).

## Materials and Methods

### Sequence collection

I obtained the SSURef 16S rRNA gene sequence database from the SILVA project (version 98; http://www.arb-silva.de) [22]. From this collection of sequences longer than 1,200 bp, I identified bacterial sequences that had an alignment quality score (ARB database field “align_quality_slv”) of 100 and were not chloroplasts, mitochondria, or suspected of being chimeric. The collection was further screened to remove sequences that had more than 5 ambiguous base positions and did not start by *E. coli* position 28 or end after position 1491. Of the remaining sequences, 13,501 sequences were unique and shared between the SILVA [22], greengenes [23], and RDP sequence collections [14]. I then generated 12 datasets from the full-length sequences using the SILVA, greengenes, and RDP alignments by extracting sub-regions of various lengths (Table 1). These regions were selected because they had already been used in publications or are amenable to the available sequencing platforms. Lane masks were generated by mapping the original mask onto the *E. coli* reference sequence and then it was applied to each of the three reference alignments [24]. In addition to the three reference alignments I generated pairwise alignments between all pairs of sequences using the Needleman-Wunsch algorithm [25] and multiple sequence alignments using MUSCLE with two iterations (maxiters = 2) and the diags option [15].

### Distance calculation methods

I implemented three sequence-based methods for calculating pairwise distances and a kmer-based distance metric. The first sequence-based method ignored any site that contained a gap; this method is implemented in the commonly used DNADIST program from the PHYLIP package [26]. The second sequence-based method counted gaps as a fifth character so that any comparison between a gap and a base was penalized as a mismatch; comparisons between two gaps were ignored. This approach asserts that every gap represents a distinct mutation. The third sequence-based method calculated distances by only penalizing a string of gaps as one mismatch [2]. This approach asserts that a gap, of any length, represents a single mutation. Distances were not corrected for multiple substitutions to simplify analysis of the data. Furthermore, some distances were so large that when they were corrected, they yielded undefined values. Distances were calculated as implemented in the mothur software package with precision to 0.0001 [27]. Finally, kmer-based distances were calculated between pairs of unaligned sequences based on their 7-base kmer profiles [28].

### Distance analysis

Pairwise distances were compared using a custom C++-coded program that calculated the linear regression coefficient using the origin as the intercept and the Pearson product-moment correlation coefficient [29]. Because several of the datasets did not demonstrate a linear correlation with the V19 region when the V19 pairwise distances were larger than 0.10, all regression and correlation coefficients are presented for V19 distances smaller than 0.10. Assessments of how much genetic diversity was either gained or lost represent the deviation from a slope of 1.0. The square of the Pearson product-moment correlation coefficient (i.e. R^2^) was used to quantify the fraction of the variation that was accounted for by the linear regression.

### α-diversity analysis

OTU- and phylogeny-based analyses were performed to assess the intra-sample biodiversity. Sequences were assigned to OTUs using the mothur implementation of the furthest-neighbor clustering algorithm [27]; although parallel analyses using the nearest and average neighbor algorithms yielded different α- and β-diversity values, the overall relationships observed with furthest neighbor algorithm were observed. The observed richness (i.e. the number of OTUs in a sample) of the dataset was calculated using every possible cutoff that the data could describe. Traditional neighbor-joining trees were generated using the clearcut software program and the distance matrices that were used in the OTU-based analyses [30]; however, the relaxed neighbor-joining algorithm was not used. The phylogenetic diversity of the data was calculated by summing the branch length for the entire tree [31]. Both analyses were replicated 50 times to assess the effects of randomization on α-diversity.

### β-diversity analysis

The OTU assignments and neighbor-joining trees created to study α-diversity were used to evaluate the effects of each variable on the ability to calculate β-diversity. Towards this end, I segregated the sequences to create two mock communities that shared 80% of their membership but had different structures. To create the mock communities full-length SILVA-aligned sequences were first assigned to OTUs using a furthest neighbor clustering of one gap distances with a cutoff of 0.05. Second, OTUs were randomly ordered. Third, 10% of the OTUs were assigned exclusively to the first community, another 10% were assigned exclusively to the second community, and the remaining OTUs were shared. For half of the shared OTUs, the probability of a sequence being from the first community was 0.375 and for the other half of the shared OTUs, the probability was 0.625. These probabilities were selected to simulate sampling two communities that had a Jaccard similarity index of 0.80 and Morisita-Horn Index value of 0.60. This process was repeated to create 100 simulated communities. Because the mock communities were not exhaustively sampled, it was unlikely that the measures would actually equal 0.80 and 0.60 for the Jaccard and Morisita-Horn indices. All β-diversity calculations were made using the mothur software package [27]. The same 100 partitions were used to analyze all distance calculation methods, alignments, regions, and β-diversity measures. I analyzed the effects of region and the alignment or distance calculation methods using a two-way analysis of variance. Each factor was highly significant (p≪0.001) and so I used the Tukey's honestly significant difference test for pairwise comparisons. Only those differences, which were non-significant (p>0.05) are indicated in figures. All test were performed within an OTU cutoff or UniFrac method.

## References

[1] Margulies M, Egholm M, Altman WE, Attiya S, Bader JS (2005). Genome sequencing in microfabricated high-density picolitre reactors.. Nature.

[2] Sogin ML, Morrison HG, Huber JA, Welch DM, Huse SM (2006). Microbial diversity in the deep sea and the underexplored “rare biosphere”.. Proc Natl Acad Sci U S A.

[3] Costello EK, Lauber CL, Hamady M, Fierer N, Gordon JI (2009). Bacterial community variation in human body habitats across space and time.. Science.

[4] Roesch LFW, Fulthorpe RR, Riva A, Casella G, Hadwin AKM (2007). Pyrosequencing enumerates and contrasts soil microbial diversity.. ISME J.

[5] Huber JA, Mark Welch DB, Morrison HG, Huse SM, Neal PR (2007). Microbial population structures in the deep marine biosphere.. Science.

[6] Liu Z, Lozupone C, Hamady M, Bushman FD, Knight R (2007). Short pyrosequencing reads suffice for accurate microbial community analysis.. Nucleic Acids Res.

[7] Huse SM, Dethlefsen L, Huber JA, Welch DM, Relman DA (2008). Exploring microbial diversity and taxonomy using SSU rRNA hypervariable tag sequencing.. PLoS Genet.

[8] Wang Q, Garrity GM, Tiedje JM, Cole JR (2007). Naive Bayesian classifier for rapid assignment of rRNA sequences into the new bacterial taxonomy.. Appl Environ Microbiol.

[9] Liu Z, DeSantis TZ, Andersen GL, Knight R (2008). Accurate taxonomy assignments from 16S rRNA sequences produced by highly parallel pyrosequencers.. Nucleic Acids Res.

[10] Youssef N, Sheik CS, Krumholz LR, Najar FZ, Roe BA (2009). Comparison of species richness estimates obtained using nearly complete fragments and simulated pyrosequencing-generated fragments in 16S rRNA gene-based environmental surveys.. Appl Environ Microbiol.

[11] Schloss PD (2009). A high-throughput DNA sequence aligner for microbial ecology studies.. PLoS ONE.

[12] DeSantis TZ, Hugenholtz P, Keller K, Brodie EL, Larsen N (2006). NAST: a multiple sequence alignment server for comparative analysis of 16S rRNA genes.. Nucleic Acids Res.

[13] Caporaso JG, Bittinger K, Bushman FD, DeSantis TZ, Andersen GL (2010). PyNAST: a flexible tool for aligning sequences to a template alignment.. Bioinformatics.

[14] Cole JR, Wang Q, Cardenas E, Fish J, Chai B (2009). The Ribosomal Database Project: improved alignments and new tools for rRNA analysis.. Nucleic Acids Res.

[15] Edgar RC (2004). MUSCLE: a multiple sequence alignment method with reduced time and space complexity.. BMC Bioinformatics.

[16] Sun Y, Cai Y, Liu L, Yu F, Farrell ML (2009). ESPRIT: estimating species richness using large collections of 16S rRNA pyrosequences.. Nucleic Acids Research.

[17] Schloss PD, Handelsman J (2006). Toward a census of bacteria in soil.. PLoS Comp Biol.

[18] Welch RA, Burland V, Plunkett G, Redford P, Roesch P (2002). Extensive mosaic structure revealed by the complete genome sequence of uropathogenic *Escherichia coli*.. Proc Natl Acad Sci U S A.

[19] Felsenstein J (2004). Inferring phylogenies.

[20] Smith AD, Lui TW, Tillier ER (2004). Empirical models for substitution in ribosomal RNA.. Mol Biol Evol.

[21] Tillier ER, Collins RA (1998). High apparent rate of simultaneous compensatory base-pair substitutions in ribosomal RNA.. Genetics.

[22] Pruesse E, Quast C, Knittel K, Fuchs BM, Ludwig W (2007). SILVA: a comprehensive online resource for quality checked and aligned ribosomal RNA sequence data compatible with ARB.. Nucleic Acids Res.

[23] DeSantis TZ, Hugenholtz P, Larsen N, Rojas M, Brodie EL (2006). Greengenes, a chimera-checked 16S rRNA gene database and workbench compatible with ARB.. Appl Environ Microbiol.

[24] Lane DJ, Stackebrandt E, Goodfellow M (1991). 16S/23S rRNA sequencing.. Nucleic Acid Techniques in Bacterial Systematics.

[25] Needleman SB, Wunsch CD (1970). A general method applicable to the search for similarities in the amino acid sequence of two proteins.. J Mol Biol.

[26] Felsenstein J (1989). PHYLIP – Phylogeny Inference Package.. Cladistics.

[27] Schloss PD, Westcott SL, Ryabin T, Hall JR, Hartmann M (2009). Introducing mothur: Open-source, platform-independent, community-supported software for describing and comparing microbial communities.. Appl Environ Microbiol.

[28] Edgar RC (2004). MUSCLE: multiple sequence alignment with high accuracy and high throughput.. Nucleic Acids Res.

[29] Sokal RR, Rohlf FJ (1995). Biometry: the principles and practice of statistics in biological research.

[30] Sheneman L, Evans J, Foster JA (2006). Clearcut: a fast implementation of relaxed neighbor joining.. Bioinformatics.

[31] Faith DP, Lozupone CA, Nipperess D, Knight R (2009). The cladistic basis for the phylogenetic diversity (PD) measure links evolutionary features to environmental gradients and supports broad applications of microbial ecology's “phylogenetic beta diversity” framework.. Int J Mol Sci.

[32] Eckburg PB, Bik EM, Bernstein CN, Purdom E, Dethlefsen L (2005). Diversity of the human intestinal microbial flora.. Science.

[33] Dethlefsen L, Huse S, Sogin ML, Relman DA (2008). The pervasive effects of an antibiotic on the human gut microbiota, as revealed by deep 16S rRNA sequencing.. PLoS Biol.

